# Ena Proteins Respond to PacC-Mediated pH Signaling Pathway and Play a Crucial Role in Patulin Biosynthesis

**DOI:** 10.3390/jof9080806

**Published:** 2023-07-30

**Authors:** Ruiling Zhuo, Yong Chen, Mengyang Xing, Zhanquan Zhang, Shiping Tian, Boqiang Li

**Affiliations:** 1 Key Laboratory of Plant Resources, Institute of Botany, Chinese Academy of Sciences, Beijing 100093, China; 2 China National Botanical Garden, Beijing 100093, China; 3 University of Chinese Academy of Sciences, Beijing 100049, China; 4 Key Laboratory of Post-Harvest Handling of Fruits, Ministry of Agriculture, Beijing 100093, China

**Keywords:** Ena family, fruit, mycotoxin, *Penicillium expansum*, blue mold

## Abstract

*Penicillium expansum* is a main producer of patulin that causes severe postharvest decay and food safety issues in the fruit industry. Development, pathogenicity, and patulin production of *P. expansum* are strongly influenced by the PacC-pH signaling pathway. Global transcription factor PacC regulates various fungal biological processes through a complicated molecular network. In the present study, three Ena family genes (*PeEnas*), *PeEnaA*, *PeEnaB*, and *PeEnaC*, as important downstream targets of PePacC, were identified in *P. expansum*. Deletion of *PeEnaA*, *PeEnaB*, and *PeEnaC* showed little effect on mycelial growth under alkaline or high salinity conditions, but double and triple deletion of these genes impaired the virulence of *P. expansum* on apple fruit. Notably, patulin biosynthesis of *P. expansum* was distinctly inhibited in the deletion mutants of *PeEnas*. *PeEnas* regulated expressions of the patulin gene cluster, *AP1*, *CreA*, *Sge1*, and *Hog1* at the transcriptional level and played roles in maintaining membrane potential. Overexpression of *PeEnaC* in Δ*PePacC* restored the patulin production defect of Δ*PePacC*. Our results indicated that, as downstream targets of PePacC, the PeEna family proteins play a crucial role in patulin biosynthesis in *P. expansum*.

## 1. Introduction

*Penicillium expansum*, a saprophytic phytopathogen, infects numerous fruit and vegetable hosts and causes severe blue mold rot. It also contaminates hosts with mycotoxin patulin, which causes food safety issues [1]. Understanding the regulatory mechanisms of pathogenicity and patulin biosynthesis will lay the foundation for the management of blue mold [2].

As one of the most important environmental factors, ambient pH significantly affects pathogen development and pathogenicity [3]. *P. expansum* can survive over a broad range of pH, with pH 4.0–5.0 being a conducive condition for spore germination and mycelial growth [4]. To sense and respond to ambient pH, fungi evolve a fungal-specific Rim/Pal signaling pathway to modulate gene expression through the activation of a key transcription factor PacC [5]. In *Aspergillus nidulans*, PacC was initially identified in three forms: PacC^72^, PacC^53^, and PacC^27^ [6]. Among them, PacC^27^ is considered the active form and is produced by the two proteolytic cleavages of the entire length form, PacC^72^. It contains three Cys_2_His_2_ zinc finger structures, and the core binding motif is 5′-GCCARG-3′ [7]. PacC regulates a variety of biological processes as a global transcription factor in fungi [8]. In *P. expansum*, PePacC was required for virulence, patulin production, conidiation, and vegetative growth [9]. PacC activates multiple alkaline-expressed genes and represses acid-expressed genes involved in transport, secondary metabolism, and cell wall degradation under neutral to alkaline conditions in *P. expansum* [9,10,11].

Ambient pH directly affects the charge of inorganic or organic acid ions. To maintain optimal cation homeostasis, cells employ diverse ion transporters. Upregulation of transporters may restore cation homeostasis in fungi at varying pH conditions [12,13,14]. The fungal cation pump is a large superfamily of plasma membrane P-type ATPases divided into five families (Types I-V) [15,16]. The Ena family proteins (Enas), corresponding to typical P-type ATPases of Group IID, couple ATP hydrolysis to transport cations against electrochemical gradients. The Ena ATPases have been recognized to be present in bryophytes, protozoa, and fungi but not in flowering plants [17,18]. Ena1/2 was originally named in *Saccharomyces cerevisiae* for Latin exitus natru (sodium exit) and was shown to mediate cellular tolerability to Na^+^, Li^+^, and alkaline pH [19]. In *S. cerevisiae*, Ena1 plays the dominant role in Na^+^ export [18]. In *A. nidulans*, three Ena orthologues (EnaA, EnaB, and EnaC) were identified, of which EnaA and EnaB were necessary in response to ions and alkaline pH, and EnaC was a putative pseudogene [20]. Enas were regulated by PacC/Rim101 pathway, Crz1 pathway, nutrient availability, and HOG pathway at transcriptional or post-transcriptional levels to adapt to high pH and salt stress [13,17,20]. PacC/Rim101 pathway was significantly involved in regulating the gene expression of *Enas* in response to alkaline pH stress in *S. cerevisiae* and *A. nidulans* [21].

Moreover, Enas significantly influenced the virulence of some fungal pathogens. The absence of Ena1 decreased virulence in pathogenic fungi, including mammalian pathogens *Cryptococcus neoformans* [22], insect pathogens *Beauveria bassiana* [23], and *Metarhizium acridum* [14]. However, functional studies of Enas in phytopathogens have rarely been reported. The aim of the present study is to investigate the functions of Enas in development, pathogenicity, and mycotoxin production in *P. expansum*. Regulation of transcriptional factor PePacC on *Enas* genes was also explored. Three orthologues (*EnaA*, *EnaB*, and *EnaC*) of the PeEna family (PeEnas) were identified in *P. expansum*. *PeEnas* were found in response to ambient pH and were directly regulated by the PePacC. *PeEnas* were involved in mycelial growth under alkaline or high salinity conditions and virulence on apple fruit. Particularly, the crucial role of PeEnas in patulin biosynthesis was revealed for the first time. 

## 2. Materials and Methods

### 2.1. Fungal Strains and Culture Conditions

*P. expansum* T01 strain isolated from infected apple fruit was taken as wild-type (*WT*) throughout this study [24]. Δ*PePacC* was constructed in our previous study [9]. The strains were cultured on potato dextrose agar (PDA) medium under dark conditions for 7–10 d at 25 °C. The conidia were collected and counted using the automated cell counter (Countstar IY1200, Shanghai, China).

### 2.2. Phylogenetic Relationships and Conserved Domain Analysis

The amino acid sequences of EnaA (CBF71175), EnaB (CBF85251), and EnaC (CBF79858) in *A. nidulans* were used as bait for PeEnaA (PEG01338), PeEnaB (PEG09496), PeEnaC (PEG10401) in *P. expansum*. The initial protein sequences of Ena homologous from other fungi were obtained from the NCBI database (http://www.ncbi.nlm.nih.gov/ (accessed on 12 August 2021)) by BLASTP. Representation of domain organization and extension of PeEnaA, PeEnaB, and PeEnaC were based on Pfam databases (http://pfam-legacy.xfam.org/ (accessed on 12 August 2021)). Multiple protein sequences mentioned above were aligned using Clustal W. With MEGA 7, a Neighbor-Joining (NJ) tree was constructed, and 1000 bootstrap replicates were performed.

### 2.3. Mutant Generation and Complementation

*P. expansum* transformation was performed by *Agrobacterium tumefaciens*-mediated transformation method (ATMT) [24]. The hygromycin phosphotransferase gene *hph* was applied as a resistance marker for single gene deletion, the neomycin resistance gene *neo* was used to construct double gene deletion and complementary strains, and the nourseothricin resistance gene *nat* was used to construct triple gene deletion strains. Homologous sequences on both sides of the target gene (5′ flanking and 3′ flanking) were amplified from DNA in the genome and inserted into multiple cloning sites of the modified pCAMBIA1300 vector with *HPH*, *NEO*, or *NAT*, respectively. The primers used for gene replacement and mutant identification are listed in Table S1. Positive transformants of *PeEnaA*, *PeEnaB*, and *PeEnaC* were screened by PCR assay and further confirmed by Southern blotting assay for single gene deletion strains (Figure S1). Full-length *PeEnaA*, *PeEnaB*, and *PeEnaC* fragments were transformed into deletion mutant strains with pCAMBIA1300-NEO in constructing complement strains.

### 2.4. Chromatin Immunoprecipitation Assay

To test whether PacC directly controls *Enas* expression, chromatin immunoprecipitation (ChIP) was performed using an antibody to PacC-GFP [25,26]. Mycelia from *WT-GFP* and Δ*PePacC::PePacC-GFP* mutant strains were collected and immersed in 1% formaldehyde for 10 min under a vacuum. Genomic DNA and protein cross-linking were performed in nuclear extraction buffer. A final concentration of 0.125 M glycine was added to the reaction system for 5 min to stop the cross-linking reaction. The fixed material was collected for nuclei extraction, as described by Wang et al. (2021) [27]. Enriched nuclei were sonicated by fragmenting the nuclear membrane and cutting gDNA to an average size of 500–1000 bp. A portion of the supernatant was set aside and reversed, cross-linked as input DNA. The remaining fraction was used as immunoprecipitation (IP) by incubating the anti-GFP antibody (ab290, dilution 1:500) with pre-blocked Dynabeads™ Protein G (Invitrogen; 10003D) overnight at 4 °C, followed by incubating chromatin samples with the antibody for 4 h, and subsequently with low salt buffer, high salt buffer, lithium chloride buffer, and TE buffer to wash the magnetic beads. IP complexes were then eluted from the magnetic beads with freshly prepared elution buffer and reversed cross-linking. The samples were then separated from the magnetic beads by elution and reverse cross-linking. IP DNA was extracted by the TIAN Quick Midi Purification Kit (Tiangen; DP204). The PacC binding sites (5′-GCCARG-3′ containing elements) in the promoters of the Ena genes were analyzed with SnapGene Viewer version 6.0.2 (http://www.snapgene.com (accessed on 29 November 2021)). Regions A and B of each gene were selected as representative regions for chromatin immunoprecipitation with qPCR (ChIP-qPCR). Specific primers were designed to amplify promoter regions surrounding 5′-GCCARG-3′ containing elements of immunoprecipitated DNA. The relative enrichment of each gene was calculated with quantitative PCR determination and normalization of IP samples to input [27].

### 2.5. Phenotypic Analysis

Phenotypic analysis was based on Chen et al. (2018) and Xu et al. (2023) methods with minor modifications [9,25]. All strains were cultured on a minimal medium (MM) and adjusted to different pH values (pH 5, pH 8) using citrate–phosphate buffer (Table S2). The colony diameters of the strains were measured by the crossover method using Vernier calipers, and the colony morphology was recorded by photographs. The virulence assay was performed on apple fruit (*Malus domestica* cv. Fuji). Four wounds (2 mm in diameter and 5 mm in depth) were placed uniformly on the equator of each apple fruit. 5 μL of conidial suspension (2 × 10^5^ conidia mL^−1^) was transferred to each wound. Inoculated fruit were kept at a constant temperature of 25 °C in high humidity. Lesion diameters were measured every 2 d. To determine patulin production, 1 µL of conidial suspension (10^6^ conidia mL^−1^) of the described strain was inoculated onto a PDA medium pre-covered with cellophane sheets (square with 1 cm sides). After 24 h of incubation, the cellophane sheets with mycelia were transferred and floated on 1 mL Czapek yeast extract (CY) medium buffered at pH 3, pH 5, and pH 8 with citrate–phosphate buffer (Table S2) for 36 h on a 24-well cell culture plate at 25 °C. Mycelia were collected for RNA extraction, and filtrates were collected for patulin determination using HPLC. HPLC detection was performed according to Li et al. [2]. The mobile phase consisted of acetonitrile and water, with a flow rate of 1 mL min^−1^ (10:90, *v*/*v*), in isocratic elution mode, and the detection wavelength was 276 nm.

### 2.6. Reverse Transcription and Quantitative PCR Analysis

Total RNA was extracted from aspirated mycelia using TRNzol universal reagent (Tiangen; DP424). The quality of RNA was assessed by utilizing 1% agarose gel electrophoresis and staining with StarStain Red Plus Nucleic Acid Dye (GenStar, China, E110-01). Moreover, the OD260/OD280 ratio of extracted RNA was quantified between 1.8 and 2.0 using a NanoDrop N-1000 spectrophotometer (NanoDrop Technologies, Wilmington, DE, USA). Reverse transcription and quantitative PCR (RT-qPCR) were carried out as previously described [24]. The data obtained were evaluated by the ΔΔCt method, using the β-tubulin gene as an internal reference. A summary of qPCR primers is provided in Table S3. A heatmap showing expression changes was generated by TBtools-II (Toolbox for Biologists) v1.120 (https://github.com/CJ-Chen/TBtools (accessed on 2 July 2022)).

### 2.7. Membrane Potential Assay

To analyze the fungal membrane potential, protoplasts were first prepared. The conidial suspension of the indicated strains in CY (15 mL, 5 × 10^7^ conidia mL^−1^) was shaken for 20 h at 25 °C and the germinating mycelia were incubated in enzymatic digestion buffer (0.8 M MgSO_4_, 1 % *w*/*v* Lysing Enzymes from Trichoderma (L1412, Sigma), 0.1 % *w*/*v* Snailase (S8280, Solarbio, Beijing, China)) for 2 h with gentle shaking (100 rpm) in dark. Protoplasts were collected by centrifugation for 5 min (1500× *g*) and then shifted to pH 5 or pH 8 conditions and continually shaken (100 rpm) for another 1 h. 2 mM bis (1,3-dibutylbarbituric acid) trimethine oxonol (DiBAC4(3), Invitrogen, Carlsbad, CA, USA) was added to the sample and incubated for 10 min at 4 °C in the dark. Fluorescence was examined using a confocal laser scanning microscope with 488 nm excitation and 509 nm emission using a confocal Zeiss 980 laser scanning microscope with Elyra7 (Zeiss, Oberkochen, Germany) [28,29]. Images and fluorescence measurements of the confocal data were captured with ZEISS ZEN 3.2 (blue edition) software (Zeiss, Oberkochen, Germany) under the same parameters.

### 2.8. Subcellular Localization of EnaC

Subcellular localization of PeEnaC proteins was observed as previously described [2], and FM4-64 (Thermo Fisher Scientific, Waltham, MA, USA) was used to stain the plasma membrane. Briefly, 5 × 10^7^ conidia mL^−1^ were incubated in CY medium for 15 h. Pre-chilled FM4-64 (1 mg mL^−1^ master mix, 1:40 dilution) was added to the 50 µL system, and samples were incubated on ice for 15–20 min, followed by confocal imaging. 488 nm/540 nm and 516 nm/640 nm excitation/emission wavelengths were used to detect the fluorescence of GFP and FM4-64, respectively.

### 2.9. Statistical Analysis

The software SPSS version 20.0 (SPSS Inc., Chicago, IL, USA) was used. Differences among multiple groups of means were analyzed by one-way ANOVA followed by Duncan’s multiple range test. The significance was considered when *p* < 0.05. For comparisons in gene expression of *PeEnaA*, *PeEnaB*, and *PeEnaC* between pH 3 and 8, and DNA fragments enrichment, a Student’s *t*-test was used.

## 3. Results

### 3.1. Sequence Features of Ena ATPases in P. expansum

A phylogenetic evolutionary tree was constructed to analyze the Ena homologs of six species, including *P. expansum*, *A. nidulans*, *B. bassiana*, *M. acridum*, *Colletotrichum gloeosporioides*, and *S. cerevisiae*. Conserved domains of Ena homologs were further analyzed to denote their roles as P-type ATPases. 

**Figure 1:**
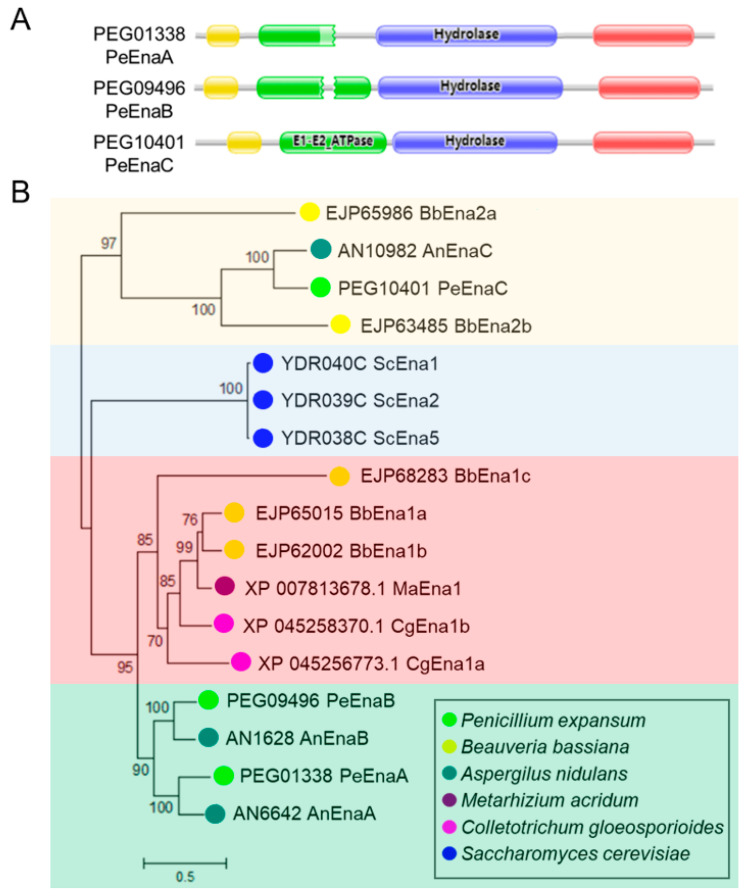
Functional conserved domain and phylogenetic tree analysis of Ena family proteins. (**A**). Functional conserved domain analysis of Ena family via Pfam database. The cation-ATPase-N (pfam00690) domain is marked in red, the E1-E2_ATPase (pfam00122) domain is marked in green, the halo acid dehalogenase-like hydrolase (HAD) (COG4087. pfam00702) domain is marked in blue, and Cation_ATPase_C (pfam00689) domain is marked in yellow. (**B**). Neighbor-Joining phylogenetic analysis of Ena family proteins in *P. expansum* and five other fungal species. MEGA 7 was used to construct the phylogenetic tree. Bootstrap values (1000 replicates) are shown for each branch. Enas of *P. expansum* are indicated in bright green.

The PeEna family has four conserved structural domains, namely the Cation_ATPase_N domain (I) (pfam00690), the E1-E2_ATPase domain (II) (pfam00122), the haloacid dehalogenase-like hydrolase domain (III) (pfam00702), and the Cation_ATPase_C domain (IV) (pfam00689) (Figure 1A). As shown in Figure 1B, the evolutionary tree constructed based on PeEna protein and its homologs was divided into four groups, with the PeEnas in *P. expansum* being most closely related to orthologous proteins in *A. nidulans* (Figure 1B). PeEna homologous proteins were predicted to have ten transmembrane regions and be localized in the plasma membrane using the TOPCONS web server (Figure S2).

### 3.2. PePacC Directly Binds to the Promoter Regions of PeEnas and Activates Transcription

**Figure 2:**
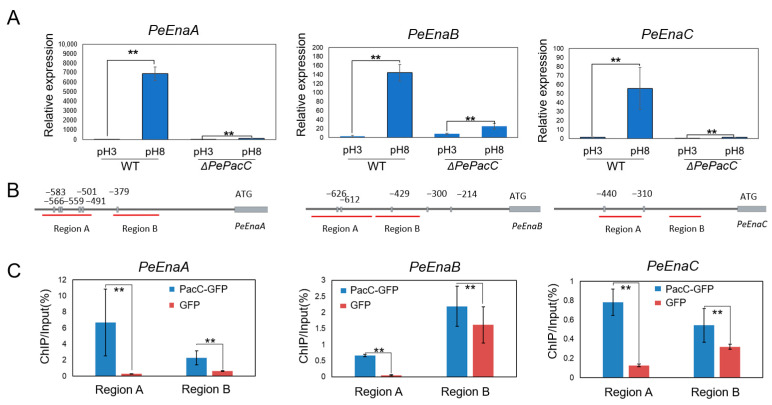
PePacC binds to the *PeEna* genes’ promoter and activates their transcription. (**A**). RT-qPCR analysis of the expression patterns of the *PeEnas* in *WT* and Δ*PePacC* at pH 3 and pH 8. Error bars represent the standard deviation of three replicates. Asterisks (**) indicate significant differences according to Student’s *t*-test (*p* < 0.01). (**B**). Analysis of the promoter regions of PacC target genes. Boxes represent elements of PacC binding motif 5′-GCCARG-3′, and numbers indicate the position of these motifs relative to the translation start site. Red lines with capital letters represent regions used for ChIP-qPCR. (**C**). ChIP-qPCR detection of the percentage of DNA fragments enriched by anti-GFP antibody in specific regions of *PeEnaA*, *PeEnaB*, and *PeEnaC* relative to input DNAs. Error bars represent standard deviation of three replicates. Asterisks (**) indicate significant differences according to Student’s *t*-test (*p* < 0.01).

As shown in Figure 2A, the expression of three *PeEnas* was up-regulated at pH 8. The relative expression levels of *PeEnaA*, *PeEnaB*, and *PeEnaC* were extensively increased by 7285-, 51-, and 41-fold in *WT* at pH 8 compared to pH 3. The deletion of PePacC markedly suppressed the expression of these genes at pH 8. Sequence analysis of 1000 bp up-stream promoter regions of *PeEna* sequences revealed the presence of 6, 5, and 2 PacC binding motif (5′-GCCARG-3′ Box) in *PeEnaA*, *PeEnaB*, and *PeEnaC* promoter regions, respectively (Figure 2B), suggesting that the expression of *PeEnaA*, *PeEnaB*, and *PeEnaC* may be regulated by PePacC. To further confirm this hypothesis, we performed ChIP-qPCR analysis to detect whether the 5′-GCCARG-3′ motif was enriched in the promoter region. The degree of PacC binding to promoters was expressed as the percentage of DNA fragments that coimmunoprecipitated with anti-GFP antibodies relative to the input DNAs. The results showed that promoter regions of *PeEnaA*, *PeEnaB*, and *PeEnaC* were significantly enriched by the anti-GFP antibody in the Δ*PePacC::PacC-GFP* strain (Figure 2C). Together, it was suggested that PePacC could bind to the promoter region and transcriptionally activate *PeEnaA*, *PeEnaB*, and *PeEnaC*.

### 3.3. PeEnas Are Involved in the Growth and Virulence of P. expansum

**Figure 3:**
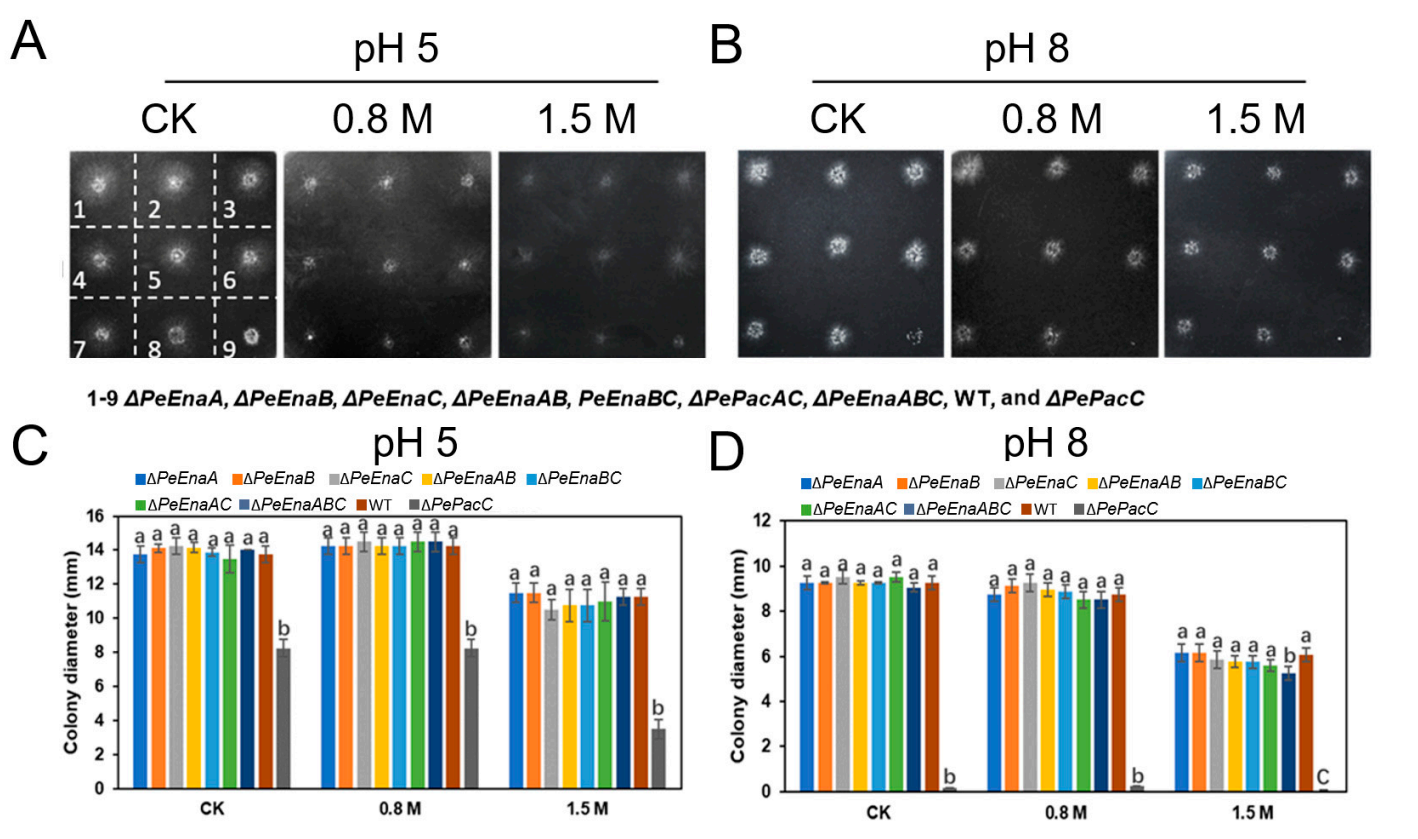
Mycelial growth of *WT*, Δ*PePacC*, and *PeEnas* knockout strains under different pH and high sodium stress of *P. expansum*. (**A**,**B**). Colony morphology of Δ*PeEnaA*, Δ*PeEnaB*, Δ*PePacC*, Δ*PeEnaAB*, Δ*PeEnaBC*, Δ*PePacAC*, Δ*PeEnaABC*, *WT*, and Δ*PePacC* (number 1–9) strains on 4 d after inoculation at MM adjusted to pH 5 and pH 8 conditions, supplemented with 0.8 M NaCl, 1.5 M NaCl. (**C**,**D**). Colony diameters of the indicated strains on MM media for 4 d at pH 5 and pH 8 conditions. Error bars represent the standard deviation of three replicates. Different letters on bars indicate significance according to One-way ANOVA followed by Duncan’s multiple range test (*p* < 0.05).

Mycelial growth among single-deletion, double-deletion, and triple-deletion strains of *PeEnaA*, *PeEnaB*, *PeEnaC*, *WT*, and Δ*PePacC* strains on MM at pH 5 and pH 8 were compared (Figure 3). The colony diameter of Δ*PeEnaABC* was reduced by about 14.3% compared to that of the WT strain when incubated at pH 8 with 1.5 M NaCl for 4 d. However, there was no significant difference between WT and *PeEnas* single or double-deletion strains under high sodium stress or alkaline pH (Figure 3C,D).

**Figure 4:**
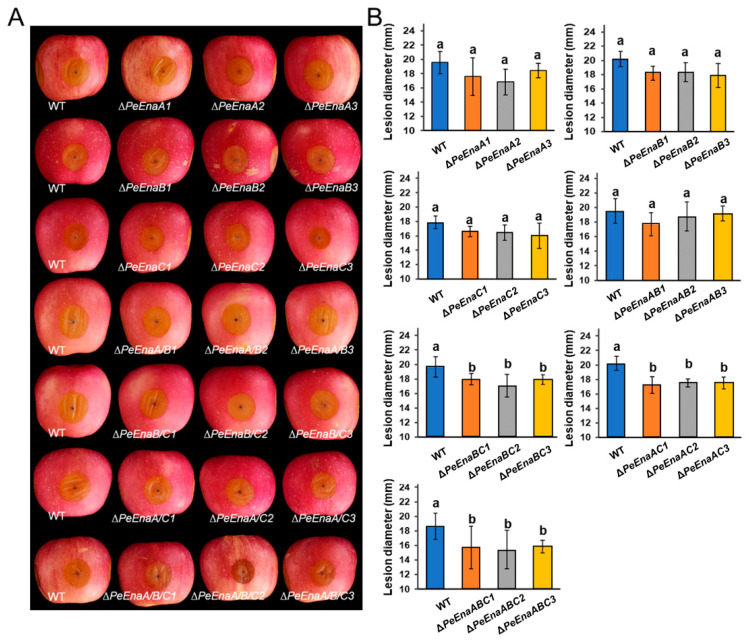
Virulence of *WT* and *PeEnas* knockout strains on apple fruit. (**A**). Disease symptoms on apple inoculated with conidia of *WT*, Δ*PeEnaA*, Δ*PeEnaB*, Δ*PeEnaC*, Δ*PeEnaAB*, Δ*PeEnaBC*, Δ*PePacAC*, and Δ*PeEnaABC* strains after inoculation. (**B**). Lesion diameters of the indicated strains after 5 d of inoculation. Error bars represent the standard deviation of three independent biological replicates. Different letters on bars indicate significance according to One-way ANOVA followed by Duncan’s multiple range test (*p* < 0.05).

In vivo, assays on apple fruit were conducted to assess the virulence of *P. expansum*. At 5 d post inoculation, lesion diameters of Δ*PeEnaBC*, Δ*PeEnaAC*, and Δ*PeEnaABC* were significantly reduced by 10.2–14.8% compared to *WT* (Figure 4A,B). 

### 3.4. PeEnas Affect Patulin Biosynthesis in P. expansum

**Figure 5:**
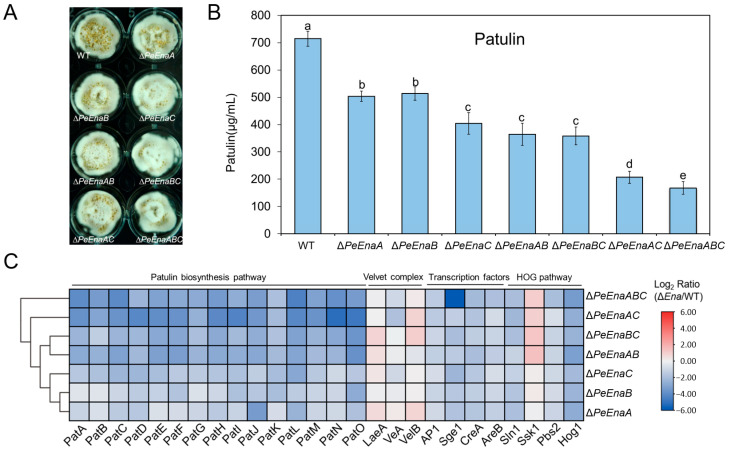
PeEnas play a vital role in patulin production. (**A**). Morphologies of the *WT*, Δ*PeEnaA*, Δ*PeEnaB*, Δ*PeEnaC*, and the relevant complementation strains after 2 d of culture in CY. (**B**). Patulin production of the indicated strains. Error bars represent the standard deviation of three independent biological replicates. Different letters on bars indicate significance according to One-way ANOVA followed by Duncan’s multiple range test (*p* < 0.05). (**C**). Heatmap showing expression changes of 15 genes in the patulin cluster (*PatA*-*PatO*), 3 coding genes in Velvet Complex, 4 coding genes of the global transcription factor, and 4 coding genes in the HOG pathway in the indicated strains. The change fold was based on the log_2_ scale of relative expression ratio and was expressed as a color gradient. Each column in the heatmap represented Δ*PeEnas* to *WT*.

The patulin production of *WT*, single-deletion, double-deletion, and triple-deletion strains of *PeEnaA*, *PeEnaB*, and *PeEnaC* was assessed (Figure 5A). Compared to *WT*, patulin production in all deletion mutants was significantly reduced (Figure 5B). Among Δ*PeEnaA*, Δ*PeEnaB*, and Δ*PeEnaC*, the patulin production in Δ*PeEnaC* was reduced the most by around 56% when compared to the *WT*. Patulin biosynthesis was further impaired in double-deletion and triple-deletion strains. Patulin production in Δ*PeEnaAC* and Δ*PeEnaABC* was reduced to 30.1% and 23.6% of *WT*, respectively (Figure 5B). In addition, expression levels of all 15 patulin cluster genes were detected by RT-qPCR assay after incubation for 2 d in the strains (Figure 5C). The results suggested that the expression levels of all genes in the gene cluster were downregulated in *PeEnas* deficient mutants. Furthermore, the expression levels of several known secondary metabolism regulators, including 3 Velvet complex components, four global transcription factors, and 4 HOG pathway members, were also evaluated. The gene expression levels of transcription factor AP1, CreA, AreB, and Sge1, as well as vital members Hog1 and Pbs2 in the HOG pathway, were significantly decreased in *PeEnas* deletion mutants in contrast to *WT* (Figure 5C).

### 3.5. PeEnaC Rescues the Defective of Patulin Biosynthesis in ΔPePacC

**Figure 6:**
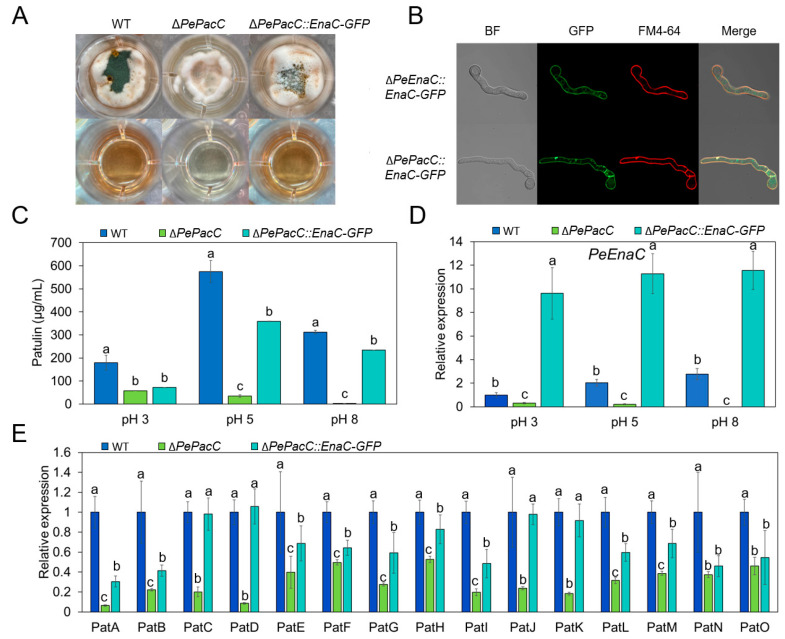
PeEnaC rescues the detective of patulin biosynthesis in Δ*PePacC*. (**A**). Morphologies of the *WT*, *∆PePacC*, and *∆PePacC::PeEnaC-GFP* in CY media at pH 5 for 2 d. (**B**). Subcellular localization of PeEnaC in Δ*PeEnaC::PeEnaC-GFP* and Δ*PePacC::PeEnaC-GFP* strains. (**C**). Patulin production of the indicated strains at pH 3, 5, and 8. (**D**). The gene expression analysis of *PeEnaC* in *WT*, Δ*PePacC*, and Δ*PePacC::PeEnaC-GFP* strains in CY media at pH 3, 5, and 8. (**E**). The gene expression analysis of patulin cluster genes of *WT*, Δ*PePacC*, and Δ*PePacC::PeEnaC-GFP* strains in CY media buffered at pH 5. Error bars represent standard deviation of three independent biological replicates. Different letters on bars indicate significance according to One-way ANOVA followed by Duncan’s multiple range test (*p* < 0.05).

*PeEnaC* as a representative of the *Enas* was selected to construct a Δ*PePacC::PeEnaC-GFP* strain. The overexpression of *PeEnaC* was validated by RT-qPCR assay (Figure 6D). Subcellular localization assay showed that PeEnaC protein was localized in the plasma membrane as TOPCONS predicted (Figure S2) in both Δ*PeEnaC::PeEnaC-GFP* and Δ*PePacC::PeEnaC-GFP* strains (Figure 6B). Patulin production of the indicated strains was further quantified. Patulin biosynthesis was significantly increased in Δ*PePacC::PeEnaC-GFP* compared to Δ*PePacC* under both pH 5 and pH 8 conditions (Figure 6C). Moreover, expression levels of all 15 patulin cluster genes were up-regulated in *PeEnaC*-complementary mutant compared to Δ*PePacC* (Figure 6E). 

### 3.6. PeEnas Involve in Maintaining Membrane Potential in P. expansum

**Figure 7:**
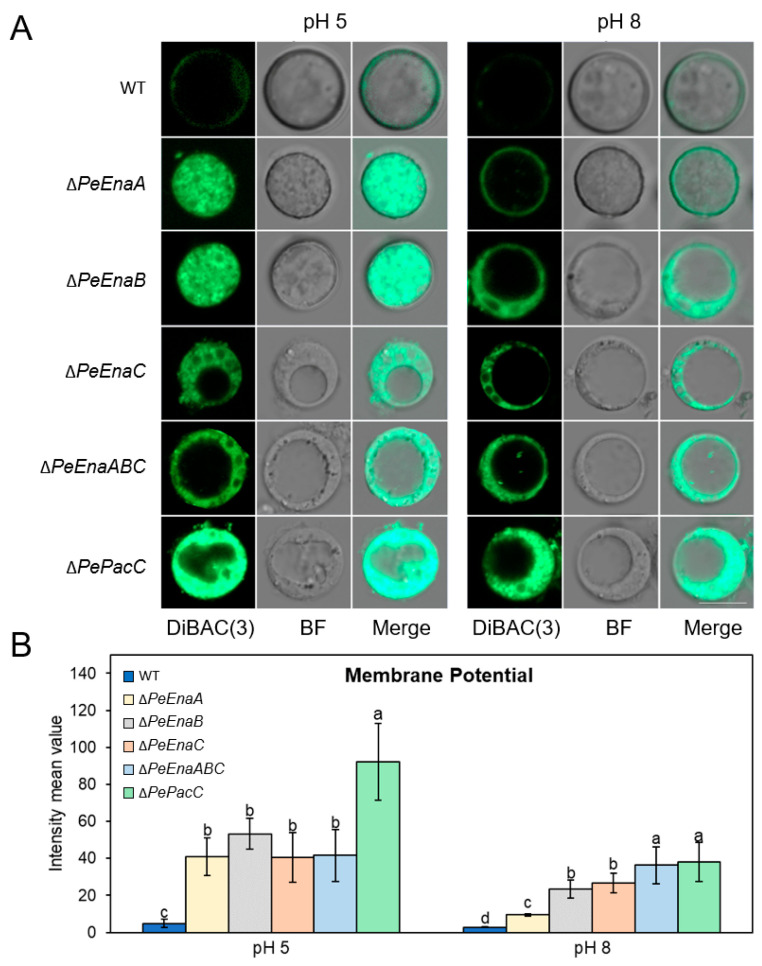
*PeEnas* involve in plasma membrane potential maintenance of *P. expansum.* (**A**). Membrane potential assay using a fluorescent indicator, DiBAC4(3), in protoplast at 20 h (control) and shifted to pH 5 or pH 8 for 1 h. Bar = 5 μm. (**B**). Fluorescence measurements were calculated with the ZEISS ZEN 3.2 (blue edition) software (Zeiss, Oberkochen, Germany). Error bars represent the standard deviation of three independent biological replicates. Different letters on bars indicate significance according to One-way ANOVA followed by Duncan’s multiple range test (*p* < 0.05).

The permeability of ions in the cell can be monitored by observing the cellular accumulation of anionic voltage-sensitive green fluorescent oxonol DiBAC4(3) [29]. DiBAC4(3) is highly voltage-sensitive and enters depolarized cells, where it binds to lipid-rich intracellular components [30]. Compared to WT, Δ*PeEnaA*, Δ*PeEnaB*, Δ*PeEnaC*, Δ*PeEnaABC*, and Δ*PePacC* strains demonstrated a higher degree of DiBAC4(3)-binding at both pH 5 and 8 conditions (Figure 7). These results indicated that the degree of depolarization was stronger in *Enas* deletion mutants and Δ*PePacC,* which has a low expression of *Enas*.

## 4. Discussion

In the present study, putative sodium ATPases of the Ena family were identified in *P. expansum* and characterized. The *P. expansum* genome encodes three PeEna ATPases, PeEnaA, PeEnaB, and PeEnaC, which is similar to that of *A. nidulans* [20]. It has been reported that AnEnaA and AnEnaB play important roles in cellular Na^+^/K^+^/H^+^ homeostasis, environmental adaptation, and virulence, while AnEnaC is a pseudogene in *A. nidulans* [20]. We found that AnEnaC did not have a complete ATPase (pfam00122) domain and HAD (pfam00702) domain (Figure S3). However, PeEnaC has a complete predicted ATPase structure (PF00122), which indicates the properties of PeEnaC were more similar to the function of typical P-type ATPases. In contrast to previous studies of Ena function, the growth of single, double, and triple-deletion mutants of *PeEnaA*, *PeEnaB*, and *PeEnaC* were unaffected in control and high salinity conditions (Figure 3). Only the triple-deletion mutant, Δ*PeEnaABC*, showed a slight decrease in colony diameter under 1.5 M Na^+^ and pH 8 conditions. *P. expansum* may have evolved other mechanisms to cope with high salt stress. In addition, we found that double and triple-deletion of *PeEnaA*, *PeEnaB*, and *PeEnaC* impaired the virulence of *P. expansum* on apple fruit, indicating that PeEnas may be involved in various biological processes.

According to the previous study, gene expression of Enas was extensively regulated to adapt to ambient pH [14,20,23,31]. PacC is the key transcription factor in the fungal-specific pH signaling pathway [8,32,33]. By analyzing the transcription pattern of the PeEnas, it was found that the gene expression levels of *PeEnaA*, *PeEnaB*, and *PeEnaC* were significantly increased at alkaline pH, and no gene transcription of any PeEnas was detected in the absence of PePacC. Further, analysis of the PePacC binding motif on the gene promoter region and ChIP-qPCR experiments demonstrated that PacC is an important positive regulator of *PeEnas* gene expression (Figure 2). This result is consistent with the previous report that Ena protein is regulated by PacC in *A. nidulans* (*AnEnaA* and *AnEnaB*), *F. graminearum* (*FgENA5*), and *A. fumigatus* (*AfEna1*) [20,34,35]. Nevertheless, Rim101(PacC) does not interact directly with the ENA1 promoter in *S. cerevisiae* but instead acts as a repressor of Nrg1 expression, which directly represses the ENA1 transcription [17]. The contrary conclusion between yeast and filamentous fungi may be attributed to the variable cis-elements of Ena orthologues generated through evolution. Fungi have evolved to use various sensors and signaling pathways to regulate cation pump expression as each fungus is exposed to different cation concentrations [35].

Few studies have been reported on secondary metabolism regulated by Ena ATPase in fungi. Interestingly, our data demonstrated that biosynthesis of vital mycotoxin in *P. expansum*, patulin, was distinctly decreased by the single deletion of three *PeEnas* (Figure 5B). Double and triple-deletion of *PeEnaA*, *PeEnaB*, and *PeEnaC* further impaired patulin production, indicating that the three PeEna family genes have a synergistic effect in regulating patulin biosynthesis. Analysis of gene expression on the patulin gene cluster suggested that PeEnas could regulate patulin biosynthesis at the transcriptional level (Figure 5C). In addition, the expressions of several well-known regulators, *AP1*, *CreA*, *Sge1*, and *Hog1* genes, were significantly downregulated in *PeEnas* deletion mutants compared to *WT*, while global Velvet complex transcription factors were not affected. In fungi, AP1-like bZIP factor AP1 regulates oxidative stress-responsive genes and is involved in secondary metabolism [36,37]. Hog1 functions in oxidative stress tolerance and is involved in trichothecene biosynthesis [38,39]. CREA ensures preferential glucose utilization by blocking the expression of other genes required for carbon source metabolism and is a transcriptional repressor of the carbon catabolite (CCR) [40]. Loss of function creA strains of *P. expansum* do not produce patulin on apple fruit [41]. Sge1 is a homologous protein of Wor1 and is involved in lifestyle switching, effector expression, and regulation of secondary metabolite biosynthesis [42]. The results suggested that PeEnas may modulate patulin biosynthesis through a broad network in *P. expansum*.

PacC, as a globally regulated transcription factor, not only mediates the pH signaling pathway but is also involved in the regulation of mycotoxin biosynthesis [8]. In *A. nidulans*, a PacC binding motif is present in the promoter region of the aflatoxin biosynthetic pathway-specific transcription factor AFLR and key enzyme IpnA encoding genes [43]. In *P. expansum*, patulin production and expression of the biosynthetic cluster were significantly down-regulated in the Δ*PePacC* strain under both acidic and alkaline conditions, indicating that PePacC positively regulates the expression of the biosynthetic gene cluster, thereby affecting patulin production [9]. PacC binding motifs were found in the promoter regions of nine patulin cluster genes [9], indicating that PePacC may directly regulate the expression of patulin biosynthetic genes. In the present study, we found that PePacC could directly regulate the expression of *PeEnas*, while the latter affected patulin production. In the three *PeEnas*, *PeEnaC* demonstrated a stronger effect on patulin production compared to *PeEnaA* and *PeEnaB* (Figure 5B). Interestingly, *PeEnaC* complementary experiment in Δ*PePacC* strain showed that overexpression of *PeEnaC* could partly restore gene expression of the patulin cluster and patulin production of Δ*PePacC* (Figure 6), indicating that PeEnas may play important roles in the regulation of PePacC on patulin biosynthesis.

## 5. Conclusions

As P-type plasma membrane ATPases, Ena family proteins are important in environmental adaptation in fungi. In the present study, three Ena family genes, *PeEnaA*, *PeEnaB*, and *PeEnaC*, were identified in *P. expansum*. All the genes responded to ambient pH and were directly regulated by PePacC, the key transcription factor of the fungal pH signaling pathway. PeEnas have little effect on mycelial growth under alkaline or high salinity conditions but are involved in virulence in fruit and maintenance of cell membrane potential. Notably, a crucial role of PeEnas in regulating the biosynthesis of secondary metabolites, patulin, was reported for the first time. PeEnas affect gene expressions of patulin cluster, AP1, CreA, Sge1, and Hog1, and play a function in the regulation of PePacC on patulin biosynthesis. The PeEna proteins may be used as potential targets for patulin contamination control. In addition, our results provide new insights for elucidating the complicated regulatory network of the global transcription factor PacC.

## Data Availability

Not applicable.

## Supplementary Materials

The following supporting information can be downloaded at: https://www.mdpi.com/article/10.3390/jof9080806/s1, Figure S1: Knockout of *PeEnaA*, *PeEnaB*, and *PeEnaC* in *P. expansum*; Figure S2: The transmembrane topology of the PeEna proteins; Figure S3: Functional conserved domain analysis of Ena family proteins via Pfam database; Figure S4: The melting curves of representative genes in this study; Table S1: The primers used for construction and identification of gene deletion, complementation and eGFP tag strains in this study; Table S2: Formulation of the citrate–phosphate buffer; Table S3: The primers used for RT-qPCR in this study.
