# Supplementary material for: Ena Proteins Respond to PacC-Mediated pH Signaling Pathway and Play a Crucial Role in Patulin Biosynthesis
Source: J Fungi (Basel). 2023 Jul 30;9(8):806. doi: 10.3390/jof9080806 (PMC10455529; doi:10.3390/jof9080806)
Supplement: Supplementary file 1 [file jof-09-00806-s001.zip › jof-2502928-supplementary.pdf]

Supplementary materials

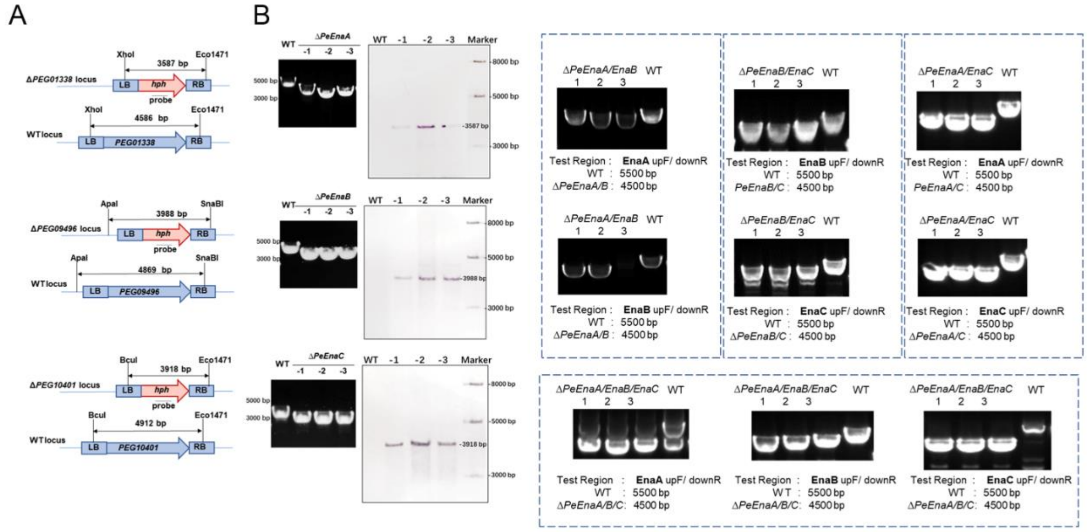

Figure S1 Knockout of *PeEnaA, PeEnaB,* and *PeEnaC* in *P. expansum*. **A.** *PeEnaA*, *PeEnaB*, and *PeEnaC* deletion strategy used by homologous recombination. **B.** PCR detection and Southern Blot of Δ*PeEnaA*, Δ*PeEnaB*, and Δ*PeEnaC* knockout mutants. WT means wild-type strain.

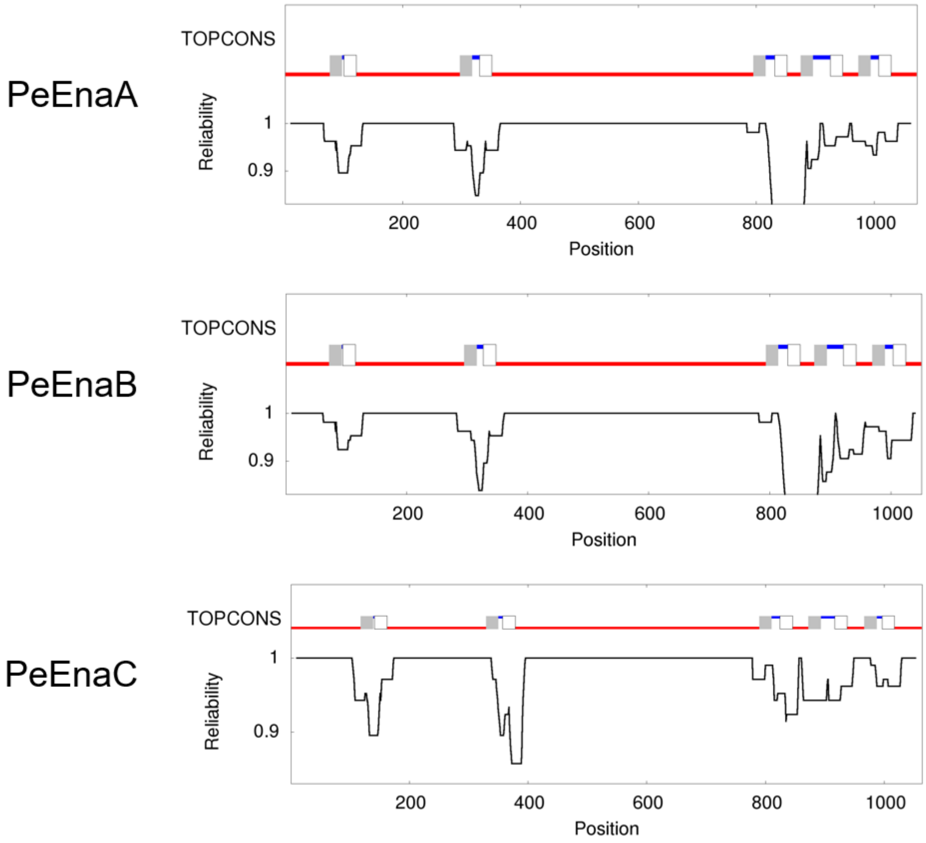

Figure S2 The transmembrane topology of the PeEna proteins. The transmembrane topology was determined by manual assertion deduced from a combination of prediction using the transmembrane topology web server TOPCONS.

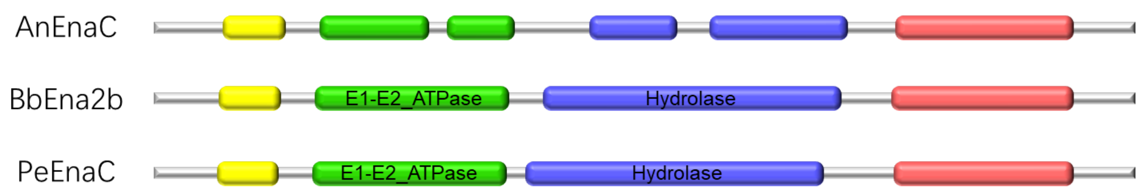

Figure S3 Functional conserved domain analysis of Ena family proteins via Pfam database. The cation-ATPase-N (pfam00690) domain is marked in red, the E1-E2_ATPase (pfam00122) domain is marked in green, the halo acid dehalogenase-like hydrolase (HAD) (COG4087. pfam00702) domain is marked in blue, and Cation_ATPase_C (pfam00689) domain is marked in yellow.

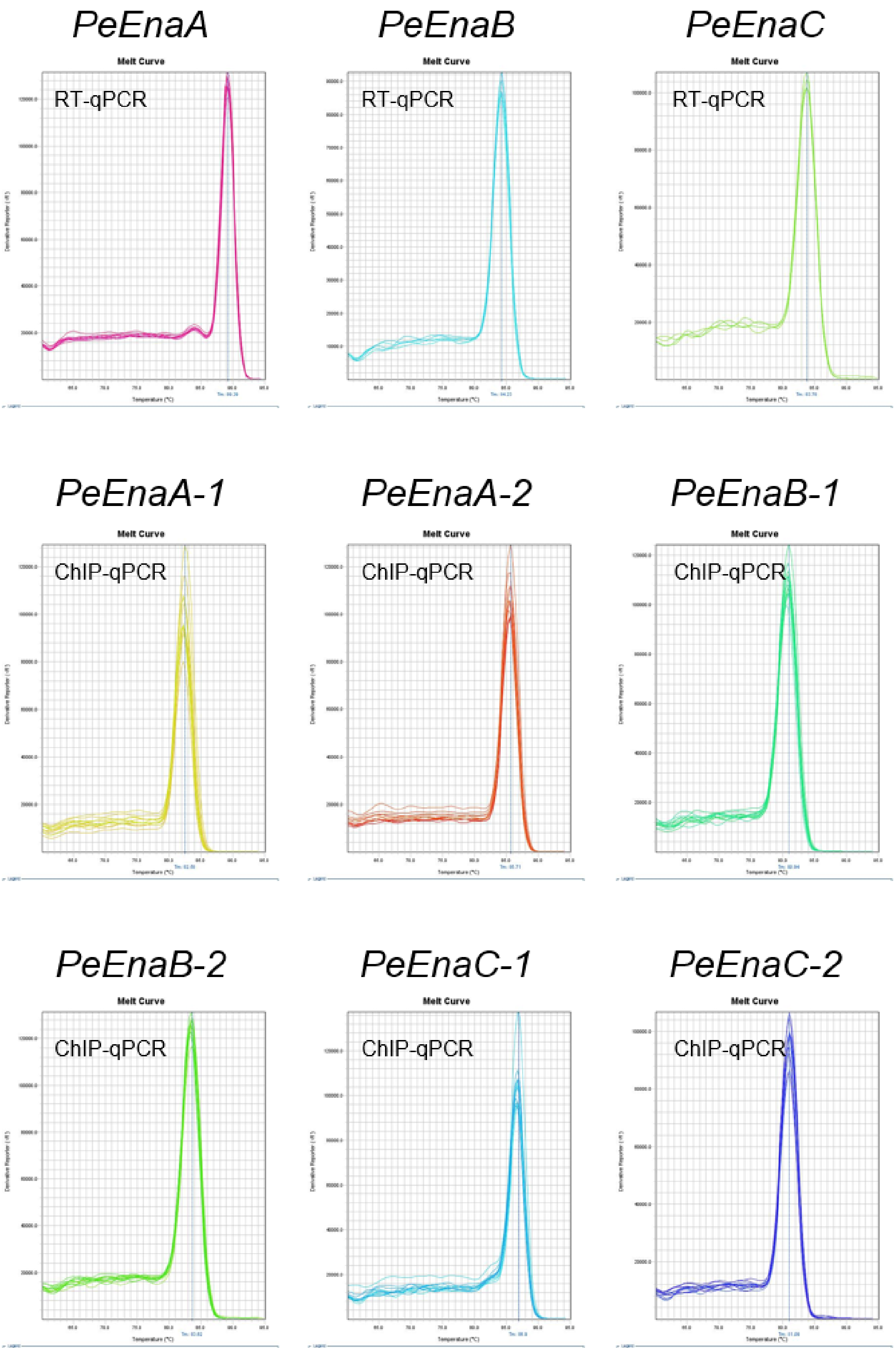
PeEnaA
Melt Curve
RT-qPCR
PeEnaB
Melt Curve
RT-qPCR
PeEnaC
Melt Curve
RT-qPCR
PeEnaA-1
Melt Curve
ChIP-qPCR
PeEnaA-2
Melt Curve
ChIP-qPCR
PeEnaB-1
Melt Curve
ChIP-qPCR
PeEnaB-2
Melt Curve
ChIP-qPCR
PeEnaC-1
Melt Curve
ChIP-qPCR
PeEnaC-2
Melt Curve
ChIP-qPCR

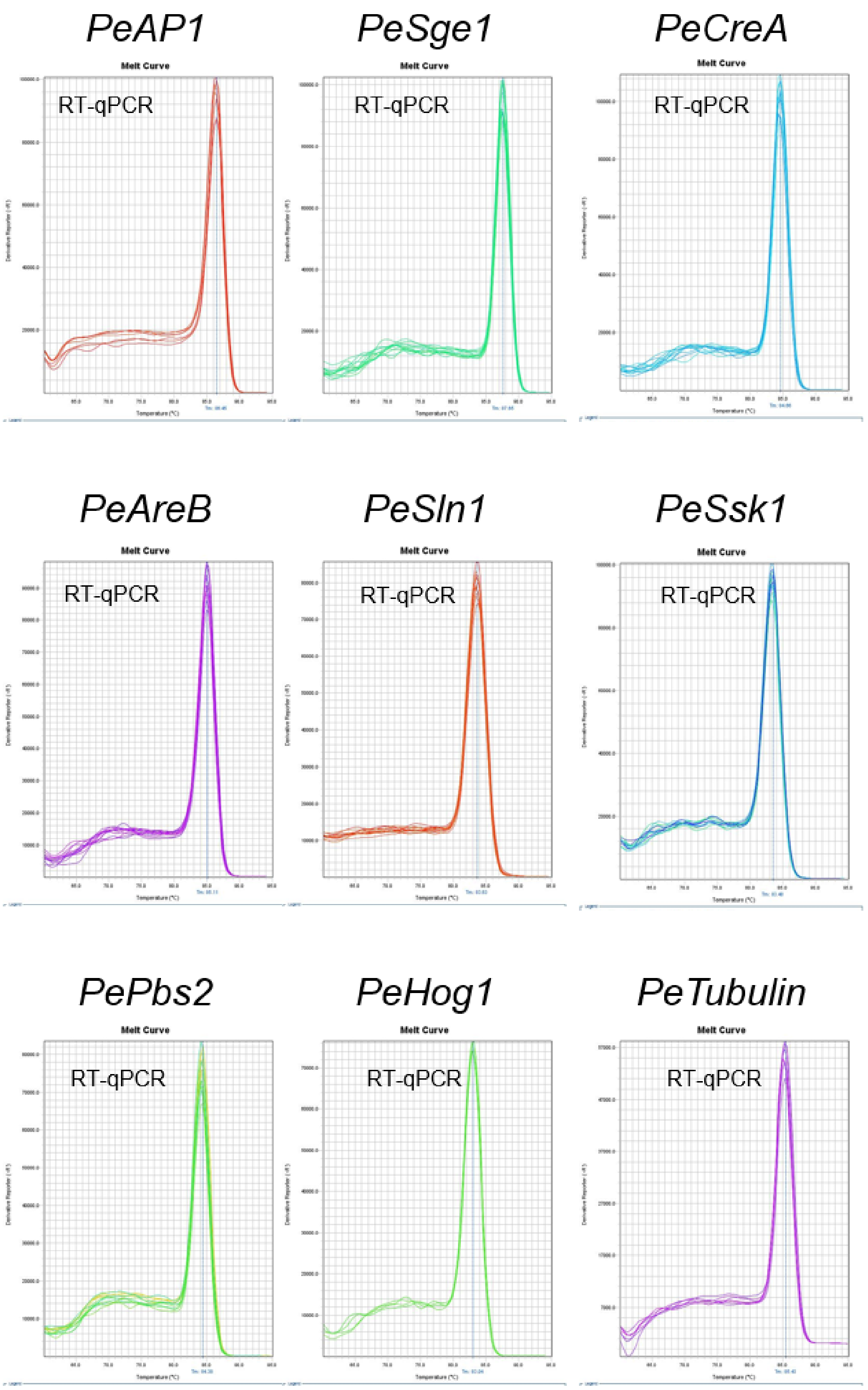

Figure S4 The melting curves of representative genes in this study.

Table S1 The primers used for construction and identification of gene deletion, complementation and eGFP tag strains in this study.

**Primer name**	**Primer sequence (5'-3')**
EnaA-up-F	GAATTCGAGCTCGGTACCCGGGTGCCCGCATGGCTGTGCGTCGTCG
EnaA-up-R	CCACAGCTGCAGGTCGACTCTAGATTCGCGATTTCGCAAATGGTAAGA
EnaA-down-F	AGTAACCATGCATGGTTGCCTAACTCAATAGATTTACTCAAATGAACAAC
EnaA-down-R	GCCAGTGCCAAGCTTCGGCGCGCCCAAATGCCGGAAGAGTTTGCAGAA
EnaC-up-F	GAATTCGAGCTCGGTACCCGGGGTTGTGTTTGAGCAAAAAAGTAGC
EnaC-up-R	CCACAGCTGCAGGTCGACTCTAGATCAATAAGATTGTAGGAAAGAAAG
EnaC-down-F	AGTAACCATGCATGGTTGCCTAACTCTGGAAAACTTGTCTGACTGAT
EnaC-down-R	GCCAGTGCCAAGCTTCGGCGCGCCTGATCATTCAACGTTGAATAAACG
EnaB-up-F	GAATTCGAGCTCGGTACCCGGGAGGAACAGCGTCTATGTTGATGCG
EnaB-up-R	CCACAGCTGCAGGTCGACTCTAGATGGATATTGTCGTTCGATGGGTAG
EnaB-down-F	AGTAACCATGCATGGTTGCCTAACTCCGAATGCTTTCCCTGGCCCAACAT
EnaB-down-R	GCCAGTGCCAAGCTTCGGCGCGCCTATGGCGATGTAATTCAACTTCGC
EnaA-test-F	GTATGCATGAAGCGCGGGGGTTTG
EnaC-test-F	ACAGGAAGATGCAGTGGGCTTCGC
EnaB-test-F	TGGCCCAGCTGAGATGAGCCATTC
HPH-F	TCACCCCCATCTCAACTCCA
HPH-R	TGCTCCATACAAGCCAACCAC
NEO-F	ATGATTGAACAAGATGGATTGC
NEO-R	TCAGAAGAACTCGTCAAGAAG
Nat-F	TCCTTCACCACCGACACCGTCTTCC
Nat-R	CGTCCGATTCGTCGTCCGATTCGTC
EnaA-Com-F	AACTCCATCACATCACAAGAGCTCTCGCAGGAAGCCCAAAAAGGCTCT
EnaA-Com-R	CTCCTCGCCCTTGCTCACTCTAGATTTGAAGATTACTTGTTCATTA
EnaB-Com-F	AACTCCATCACATCACAAGAGCTCATGTCAGACGAGAAGCCTGGCA

EnaB-Com-R	CTCCTCGCCCTTGCTCACTCTAGATTAGGGTTGCTCCGGGATCTC
EnaC-Com-F	AACTCCATCACATCACAAGAGCTCATAGAGGTAACTCCCGATGTCGA
EnaC-Com-R	CTCCTCGCCCTTGCTCACTCTAGACTTCAGTTCCACGCTTGCATTCGC

Table S2 Formulation of the citrate-phosphate buffer

pH	0.2 mol/L Na_2HPO_4（mL）	0.1 mol/L Citric acid（mL）
3	4.11	15.89
5	10.30	9.70
8	19.45	0.55

Note:

Preparation of MM media at pH 5 and 8: Prepare 2× MM medium and the above citrate-phosphate buffer, and preheat them at 80°C, respectively. Mix the medium and the buffer in a ratio of 1:1 and spread the plates.

Preparation of CY media at pH 3, 5, and 8: Prepare 2× CY liquid medium and the above citrate-phosphate buffer, Mix them in a ratio of 1:1.

Table S3 The primers used for RT-qPCR in this study.

Name	Sequence (5'-3')	Annealing temperatures (˚C)	Accession number
EnaA-RegionA	F: CTGGAGAAGTAGGTGGCTTTGAA<br>R: CTCGCCCGGTCCCCACGCGACAAT	60	PEX2_013210 (PEG01338)
EnaA-RegionB	F: ATGTGCCCAGAGTCTTTTGCGTCA<br>R: TCGATGGCGAAAGATAAGCGGCT	60	PEX2_013210 (PEG01338)
EnaB-RegionA	F: TGCTCCTTCCCAGTGTTGTTGTG<br>R: GCTGTGAACTTGCCTCGTCTTGC	60	PEX2_039950 (PEG09496)
EnaB-RegionB	F: TGGAGCAAGACTGTTGAAGTGGC<br>R: GACAGACATCGGAATCTGGTGGG	60	PEX2_039950 (PEG09496)
EnaC-RegionA	F: CCACGACTGCCTAGAAGCCAAGT<br>R: TCCGACGGGAGTGTTCAAAGATA	60	PEX2_085740 (PEG10401)
EnaC-RegionB	F: CCCTCTTAGGGTTGGAAGCAAGT<br>R: GTAGCAAGGCGGACGAAGGTGTA	60	PEX2_085740 (PEG10401)
PeLaeA	F: TCGAAGCTCTTCCGGACATG<br>R: AATGCAATGCGGTCAATCTG	60	PEX2_035880 (PEG08484)
PeVeA	F: GAAGATACTTTCGGCCTTGATGA<br>R: TCGCGATAAGCAGGATAAGGA	60	PEX2_043190 (PEG11106)
PeVelB	F: TTGTTGTCTGGGTCCGTCAA<br>R: CCTGGGATGAGGGATTCGT	60	PEX2_031850 (PEG02453)
PeAP1	F: GAGCCTTGTTGCCCAGATTGATA<br>R: GTTCACGACTACACCACCCTCA	60	PEX2_065210 (PEG01534)
PeSge1	F: ATGGAATGTACTTCGGGCTGCTT<br>R: TAGTGGGTCGTTGGATGGGATAA	60	PEX2_061550 (PEG11642)
PeCreA	F: TCACGATACTTCGCCCGACTCAA<br>R: GAAGGCACGGTCGCAAAGAGGAC	60	PEX2_022240 (PEG01401)
PeAreB	F: CTATACCCTCATAATGGCGACCAC<br>R:TTCAAGAAGAGTCCACATGCGTTG	60	PEX2_003790 (PEG07853)
PeSln1	F: CCCAGACCGACTCAAGTATCAAC<br>R:GCTATCTAGCAGAATGTCACCTCCC	60	PEX2_054790 (PEG04458)
PeSsk1	F: CGATCAATACAATTCTTCCTTGCC<br>R: GCTGGGATTGGAGTTTGAGCTTTC	60	PEX2_072810 (PEG02228)
PePbs2	F: GGAACTGGGTTATCTGCCTCGTC<br>R: TGCCCTGTTGAAGCAGCAGTATG	60	PEX2_005030 (PEG09483)
PeHog1	F: CACCGCTCTTCAAATCACCGACAG<br>R: TGCCATCAAACGGCTTCCCTATC	60	PEX2_060120 (PEG11493)
PePatA	F: AAAGGCCGGTGCATTGATC<br>R: TTGGAGGCTTTGGTGAGCAT	60	PEX2_082810 (PEG03683)
PePatB	F: GCCAGGCTATGCGATTGAGT<br>R: GCTGGAACCCTGTCCATTGT	60	PEX2_082800 (PEG03684)

PePatC	F: TCCACCTGCGAATATCCCTTA<br>R: CATCGCCAGTGCCATTTTC	60	PEX2_082790<br>(PEG03685)
PePatD	F: ATGAGATTCGTCTGCGCAAAG<br>R: CTACCCAAGCGGGATGAGATT	60	PEX2_082780<br>(PEG03686)
PePatE	F: CATTCTCATCGGGCCTGAGT<br>R: TCGAAGCTCTTCCGGACATG	60	PEX2_082770<br>(PEG03687)
PePatF	F: GCGAGTGAATTCGGCCAAT<br>R: GTCCGACCCAAAGGATGAAG	60	PEX2_082760<br>(PEG03688)
PePatG	F: CGGCCGTCTTGAAGGAAAT<br>R: CTTGCCGTAGCGGGTGAATA	60	PEX2_082750<br>(PEG03689)
PePatH	F: CATTTATCGGCGGTGTTCTGA<br>R: GATCAACGCTTGCACGATAGC	60	PEX2_082740<br>(PEG03690)
PePatI	F: GCAAACTCATTCCGCAAGGA<br>R: TGGTTCTTGCCATCGATCAC	60	PEX2_082860<br>(PEG03676)
PePatJ	F: CGCCAGACATACCGCCATA<br>R: TTTGGTCGATCGGGACTGTT	60	PEX2_082870<br>(PEG03675)
PePatK	F: GACGCTGGGCTACTGGATTG<br>R: TCGTGCGTGAGGCCAGTAT	60	PEX2_082880<br>(PEG03674)
PePatL	F: GCAGGAGATCCGTTTCAGACA<br>R: CCACTGACCGACGGTTACAAC	60	PEX2_082850<br>(PEG03677)
PePatM	F: ACCCACAGCTGCACATGGA<br>R: AGCGAGAAGAGGCGGAAGA	60	PEX2_082820<br>(PEG03681)
PePatN	F: CGTTCGATGTCGCTAGCAAA<br>R: GGCGATAATCACGTCAATTCG	60	PEX2_082830<br>(PEG03680)
PePatO	F: TCGCCTCCTGGTGTGTATCTT<br>R: AAGCGTGCCCAGTCATTCAG	60	PEX2_082840<br>(PEG03678)
β-tubulin	F: CTCCAGCTCGAGCGTATGAAC<br>R: GGCTCCAAATCGACGAGAAC	60	PEX2_027410<br>(PEG11155)
